# The syndrome of inappropriate antidiuretic hormone associated with nasal and paranasal malignant tumors

**DOI:** 10.1007/s00405-023-08347-5

**Published:** 2024-01-02

**Authors:** Shoutao Dang, Shurong Zhang, Jingyang Zhao, Xinyu Li, Wei Li

**Affiliations:** Cancer Center, Beijing Tongren Hospital, Capital Medical University, Beijing, 100730 China

**Keywords:** Syndrome of inappropriate secretion of antidiuretic hormone, Nasal and paranasal tumors, Olfactory neuroblastoma, Hyponatremia

## Abstract

**Purpose:**

To investigate the clinical characteristics of the syndrome of inappropriate antidiuretic hormone (SIADH) associated with nasal and paranasal malignant tumors.

**Methods:**

Patients with locally advanced or recurrence/metastatic malignant tumors of the nasal and paranasal sinuses were included. The SIADH was diagnosed according to the diagnostic criteria. The clinical characteristics of SIADH patients were retrospectively analyzed.

**Results:**

Six patients (6/188, 3.2%) met the diagnostic criteria of SIADH, including four olfactory neuroblastoma (4/26, 15.4%), one neuroendocrine carcinoma (1/9, 11.1%), and one squamous cell carcinoma (1/63, 1.6%). Five patients (83.3%) had severe hyponatremia; however, the hyponatremia could be improved by fluid restriction or tolvaptan. Three patients' SIADH were recovered during the chemotherapy and the other three were recovered after the surgery.

**Conclusion:**

The incidence of SIADH associated with nasal and paranasal malignant tumors is relatively more common in olfactory neuroblastoma and neuroendocrine carcinoma. The hyponatremia caused by SIADH may be corrected by fluid restriction or tolvaptan, and the SIADH may be recovered through anti-tumor therapy.

## Introduction

The syndrome of inappropriate secretion of antidiuretic hormone (SIADH) associated with malignant tumor is one of the common endocrine paraneoplastic syndrome. It is a group of syndromes caused by tumor ectopic secretion that leads to the inappropriate secretion of antidiuretic hormone, and then causes water retention, increased urinary sodium excretion, dilutive hyponatremia and other clinical manifestations [1]. SIADH related hyponatremia may present as asymptomatic, severe symptoms or even life threatening depending on the serum sodium concentration [2]. The initial treatment for the hyponatremia due to SIADH is fluid restriction; however, it is not often possible in oncology patients because hydration is often required in intravenous chemotherapy applications [3]. Tolvaptan, a selective vasopressin V2-receptor antagonist, is also used to treat SIADH associated hyponatremia with good efficacy [4].

A variety of tumor sites and pathological types may cause SIADH [5], among which small cell lung cancer is the most common tumor type [6, 7], while the incidence of SIADH in other sites is very low. There are various pathological types of nasal and paranasal malignancies. Squamous cell carcinoma (SCC) is the most frequency type; however, other types such as adenocarcinoma, soft tissue sarcoma, neuroendocrine carcinoma (NEC) and olfactory neuroblastoma (ONB) are relatively more common than the other head and neck malignant tumor [8]. The studies concern about SIADH associated with nasal and paranasal malignancies are rare and mostly case reports [9].

We conducted a retrospective analysis in our single center between March 2018 and March 2023 to investigate the clinical characteristics of the SIADH associated with nasal and paranasal malignant tumors.

## Materials and methods

### Patients

Patients with locally advanced or recurrence/metastatic malignant tumors of the nasal and paranasal sinuses treated in our center from March 2018 to March 2023 were selected as research objects. The patients who met the diagnostic criteria of SIADH were included in this study. The information was collected, including: name, sex, age, pathological type, tumor stage, anti-tumor therapy regimen, efficacy of chemotherapy, time of onset of SIADH, serum sodium, urine sodium or urine osmolality at diagnosis of SIADH, treatment of SIADH, and outcome of SIADH. The information was retrospectively analyzed, and the clinical characteristics of SIADH associated with malignant tumors of nasal cavity and sinuses were explored.

### Diagnosis of SIADH

The diagnosis of SIADH included six essential criteria: serum osmolality < 275 mOsm/kg; urine osmolality > 100 mOsm/kg at some level of serum hypoosmolality; clinical euvolemia, as defined by the absence of signs of volume depletion; elevated urine sodium concentration > 30 mmol/L with normal dietary salt and water intake; absence of other potential causes of euvolemic hypoosmolality such as severe hypothyroidism, adrenal insufficiency; normal renal function and absence of diuretic intake. And there were seven supplemental criteria: serum uric acid < 4 mg/dl; serum urea < 21.6 mg/dl; failure to correct hyponatremia after 0.9% saline infusion; correction of hyponatremia through fluid restriction; fractional sodium excretion > 0.5%; fractional urea excretion > 55%; fractional uric acid excretion > 2% [10].

### Management of SIADH

When hyponatremia occurred, hypertonic saline or isotonic saline was administered according to the severity of hyponatremia and clinical symptoms. If hyponatremia was not improved by saline infusion, then urine sodium was tested. When SIADH was diagnosed, fluid restriction was initiated. Tolvaptan was added for the patients who were ineffective or unfit for fluid restriction. The treatment of hyponatremia was periodically interrupted if the serum sodium was stable. And we considered SIADH be recovered if the serum sodium maintained normal while the treatment of hyponatremia withdrew.

## Results

There were 188 patients with locally advanced or recurrence/metastatic malignant tumors of the nasal and paranasal sinuses from March 2018 to March 2023, including 63 cases of squamous cell carcinoma (33.5%), 43 cases of soft tissue sarcoma (22.9%), 26 cases of olfactory neuroblastoma (13.8%), 23 cases of adenocarcinoma (12.2%), ten cases of undifferentiated carcinoma (5.3%), nine cases of neuroendocrine carcinoma (4.8%), six cases of malignant melanoma (3.2%), four cases of lymphoma (2.1%), and four cases of others (2.1%). Six patients (6/188,3.2%) met the diagnosis criteria of SIADH, including four ONB (4/26,15.4%), one NEC (1/9,11.1%), and one SCC (1/63,1.6%). The information of the patients was shown in Table 1. All the six patients were male, aged 37–67 years old, with Ki67 index 30%-90%. There was only one patient with moderately differentiated SCC, while the other five patients were all poorly differentiated ONB and NEC. In terms of tumor staging, five patients were locally advanced and one patient was metastatic NEC.

**Table 1:** The general information of the six SIADH patients

No.	Sex	Age	Pathology type	Differentiation/grade	Ki67	Tumor stage
1	Male	63	SCC	Moderately differentiated	30%	Locally advanced
2	Male	44	ONB	Grade 3	90%	Kadish C
3	Male	55	NEC	Undifferentiated	80%	Metastatic
4	Male	37	ONB	Grade 3	75%	Kadish C
5	Male	67	ONB	Grade 3	50%	Kadish C
6	Male	35	ONB	Grade 3	40%	Kadish C

Among the six patients with SIADH, four patients (66.7%) present with synchronous SIADH when the malignant tumor was diagnosed, and two patients (33.3%) present metachronous SIADH after the first cycle of chemotherapy. Both two metachronous SIADH patients had partial response to the chemotherapy, and both SIADH recovered after two and three cycles of chemotherapy respective. The serum sodium at the time of SIADH was diagnosed ranged from 116.5 to 126 mmol/L. Five patients (83.3%) had severe hyponatremia (serum sodium < 125 mmol/L) and one (16.7%) had moderate hyponatremia (serum sodium 125–129 mmol/L). Hyponatremia could be corrected in all the six SIADH patients. One patient had his hyponatremia improved simply by fluid restriction, while the other five patients needed the additional of tolvaptan treatment. One patient with metastatic NEC applied first line chemotherapy and subsequent radiotherapy due to the good response to the chemotherapy. Five patients with locally advanced ONB and SCC utilized induction chemotherapy followed by surgery and adjuvant radiotherapy. Four patients (66.7%) had partial response (PR) base on RECIST 1.1 criteria, and the other two (33.3%) were stable disease (SD). Three (50%) patients' SIADH were recovered after the second or third cycle of chemotherapy, and the other three patients were recovered after surgery. All the three patients whose SIADH recovered during chemotherapy had partial response. However, among the three patients whose SIADH did not recover during induction chemotherapy, only one patient had partial response and the other two patients had stable disease (see Table 2).

**Table 2:** The clinical characteristics of the six SIADH patients

No.	Onset time of SIADH	Serum sodium(mmol/L)	Treatment of hyponatremia	Anti-tumor therapy	Efficacy of CT	Outcome of SIADH
1	Synchronous	123.2	Fluid restriction	Chemo → S → Radio	PR	Recover during Chemo
2	Synchronous	126	Tolvaptan	Chemo → S → Radio	SD	Recover after surgery
3	Metachronous	121	Tolvaptan	Chemo → Radio	PR	Recover during Chemo
4	Metachronous	123.4	Tolvaptan	Chemo → S → Radio	PR	Recover during Chemo
5	Synchronous	116.5	Tolvaptan	Chemo → S → Radio	SD	Recover after surgery
6	Synchronous	121.1	Tolvaptan	Chemo → S → Radio	PR	Recover after surgery

*Chemo* chemotherapy, *Radio* radiotherapy, *S* surgery

## Discussion

SIADH refers to euvolemic states of hyponatremia due to impaired water excretion resulting from nonphysiologic stimuli for arginine vasopressin (AVP) /antidiuretic hormone (ADH) production. The pathophysiology and main subtypes of SIADH are as follows [11]. Type A (40–70% of cases): “random” ADH/AVP secrete, independent of serum osmolality; Type B (20–40%): “reset osmostat”. ADH/AVP neurosecretion caused by serum Na^+^ decline, consider as “normal”; Type C (10%): ADH/AVP is not suppressed by water load; Type D (< 5%): normal ADH/AVP secrete but lower receptor threshold. There are many pathogeneses for SIADH, and the malignant tumor itself or the anticancer drugs are the common causes. Malignant tumor related SIADH is more common in the lung small cell neuroendocrine carcinoma, although it can be identified in different solid tumors. The studies that concern on SIADH associated with nasal and paranasal malignancies are rare and mostly case reports. To the best of our knowledge, our study had the largest number of nasal and paranasal malignancies related SIADH patients in single center. We hope it may provide some help in better understanding of the clinical characteristics of the SIADH.

SIADH was diagnosed in six patients with locally advanced or metastatic nasal and paranasal malignancies. Five patients (14.3%, 5/35) were ONB (4/26, 15.4%) or NEC (1/9, 11.1%), and this incidence of SIADH was similar to small cell lung cancer in a prospective single-center study (9.1%, 35/385) [12]. ONB is an uncommon malignant tumor of the nasal and paranasal arising from the olfactory epithelium. The morphology and immunophenotype of ONB overlap with other sinonasal small round blue cell tumors, such as neuroendocrine carcinoma [13]. Therefore, we speculated that SIADH may also have a high incidence in ONB and NEC in the nasal and paranasal as in the lung small cell neuroendocrine carcinoma. Only one patient with squamous cell carcinoma was moderately differentiated and Ki67 (30%), while the other five patients with ONB and NEC were all high-grade or poorly differentiated tumors and high expression of Ki67 (40–90%). This also suggested that, poor differentiation and high expression of Ki67 which is similar to the biological characteristics of small cell lung cancer, may also be the risk factors for SIADH in the nasal and paranasal.

Hyponatremia was a well-known prognostic factor in malignant patients and it had a negative influence on performance status and hospitalization [14, 15]. Hyponatremia due to SIADH was mainly severe (82.9%, 29/35) in the prospective small cell lung cancer single center study [12].Tolvaptan had been approved in the US and Europe for the treatment of hyponatremia caused by SIADH. In the study of SALT, patients with hyponatremia who received tolvaptan had significantly increased serum sodium compared with placebo (*p* < 0.0001). A subset of 110 patients with SIADH were also analyzed and showed the same results [16]. In our study, five patients had severe hyponatremia and one had moderate hyponatremia. The hyponatremia could be improved by simply fluid restriction in one patient, or by the addition of tolvaptan in the other five patients. Therefore, although severe hyponatremia was seen in a large proportion of patients, it could be improved by appropriate treatment.

Both two SIADH patients that occurred after the first cycle of chemotherapy had PR to the chemotherapy, and the SIADH recovered during the chemotherapy. However, it is difficult to determine whether these two SIADH patients were caused by tumor necrosis after chemotherapy or by chemotherapy drug itself. This clinical phenomenon also reminds us to pay attention to the diagnosis and treatment of SIADH throughout the whole chemotherapy period.

All the six patients had their SIADH recovered, three were during the chemotherapy and were all PR to the chemotherapy, the other three were after the surgery while only one was PR. We believe that SIADH can be recovered by anticancer therapy such as chemotherapy or surgery. The improvement of SIADH may be a prediction for the good response to anticancer therapy.

Since SIADH associated with nasal and paranasal malignant tumors was very rare, we conducted a mini-literature review for recent two decades in PubMed with the Medical Subject Headings (MeSH) terms “Inappropriate ADH Syndromeand” AND “Nose Neoplasms OR Paranasal Sinus Neoplasms” and their entry terms. 12 studies [9, 17–27] with 14 patients were found among which 11 studies were case report and the other one was case series (Table 3). For all the 14 patients, 11 patients were ONB and the other three were NEC, 13 patients had severe hyponatremia while the other one had moderate hyponatremia, 13 patients' SIADH recovered after anti-tumor therapy and only one patient' SIADH did not recover through palliative radiotherapy. These previous reported cases of nasal and paranasal malignant tumors related SIADH had similar clinical characteristics with our study.

**Table 3:** Previous reported cases of SIADH associated with nasal and paranasal malignant tumors

Author	Year	Study type	Age/sex	Pathology type	Serum sodium	Anti-tumor therapy	Outcome of SIADH
Nilesh Vasan [17]	2004	Case report	30 F	NEC	125 mEq/L	Chemo → Radio → S	Recover during Chemo
Lo´pez Plasencia [18]	2006	Case report	34 F	ONB	121 mM/L	Surgery	Recover after surgery
Hiroto Maeda [19]	2007	Case report	61 M	ONB	112 mmol/L	Radio	Not recover
Ma [20]	2009	Case report	66 M	NEC	118 mg/dL	Chemo	Recover during Chemo
Stacey T. Gray[21]	2012	Case series	29 M	ONB	114 mmol/L	Chemo → S → CRT	Recover after surgery
			25 F	ONB	115 mmol/L	S → Radio	Recover after surgery
			32 F	ONB	110 mmol/L	S → Radio	Recover after surgery
Ali Cemal Yumusakhuylu [22]	2013	Case report	38 M	ONB	123 mEq/L	Surgery	Recover after surgery
Anne-Sophie Sejling [23]	2014	Case report	38 M	ONB	120 mmol/L	Surgery	Recover after surgery
Zi Yang Jiang [24]	2015	Case report	10 M	ONB	109 mEq/L	Surgery	Recover after surgery
Bach [25]	2016	Case report	64 M	NEC	119 mmol/L	Chemo	Recover after Chemo
Parrilla [26]	2017	Case report	31 M	ONB	111 mmol/L	Surgery	Recover after surgery
Takafumi Nakano [27]	2017	Case report	31 F	ONB	116 mEq/L	S → CRT	Recover after surgery
Eugene Wong [9]	2019	Case report	17 F	ONB	111 mmol/L	Surgery	Recover after surgery

*Chemo* chemotherapy, Radio radiotherapy, *S* surgery, *CRT* chemoradiotherapy

Our study has some limitations. Firstly, our study did not contain survival prognostic outcome, although it might be heterogeneity due to the different pathological types and tumor stages. Secondly, this is a case series study and we still need higher quality research to confirm our result.

## Conclusion

The incidence of SIADH is low in nasal and paranasal malignancies. However, it is relatively more common in olfactory neuroblastoma and neuroendocrine carcinoma. In addition, poor differentiation and high expression of Ki67 may also be the risk factors for SIADH. SIADH may also occur during chemotherapy, so attention should be paid to manage SIADH during the whole chemotherapy period. The hyponatremia caused by SIADH may be corrected by fluid restriction or tolvaptan, and the SIADH may be recovered through anti-tumor therapy.

## Data Availability

The data underlying this article are available in the article.
